# Design and Micro-Fabrication of Focused High-Frequency Needle Transducers for Medical Imaging

**DOI:** 10.3390/s22103763

**Published:** 2022-05-15

**Authors:** Thanh Phuoc Nguyen, Jaeyeop Choi, Van Tu Nguyen, Sudip Mondal, Ngoc Thang Bui, Dinh Dat Vu, Sumin Park, Junghwan Oh

**Affiliations:** 1 Department of Mechatronics, Cao Thang Technical College, Ho Chi Minh City 700000, Vietnam; 2 Industry 4.0 Convergence Bionics Engineering, Pukyong National University, Busan 48513, Korea; eve1502@pukyong.ac.kr (J.C.); nguyentu@pukyong.ac.kr (V.T.N.); dinhdatvn96@pukyong.ac.kr (D.D.V.); tnlas030980@pukyong.ac.kr (S.P.); jungoh@pknu.ac.kr (J.O.); 3 New-Senior Healthcare Innovation Center (BK21 Plus), Pukyong National University, Busan 48513, Korea; sudip1984@pknu.ac.kr; 4 Institute of Engineering, HUTECH University, Ho Chi Minh City 700000, Vietnam; bn.thang@hutech.edu.vn; 5 Ohlabs Corporation, Busan 48513, Korea

**Keywords:** needle transducer, focused transducer, high-frequency transducer, biomedical imaging

## Abstract

In this study, we report an advanced fabrication technique to develop a miniature focused needle transducer. Two different types of high-frequency (100 MHz) transducers were fabricated using the lead magnesium niobate-lead titanate (PMN-0.3PT) and lithium niobate (LiNbO_3_) single crystals. In order to enhance the transducer’s performance, a unique mass–spring matching layer technique was adopted, in which gold and parylene play the roles of the mass layer and spring layer, respectively. The PMN-0.3PT transducer had a 103 MHz center frequency with a −6 dB bandwidth of 52%, and a signal-to-noise ratio (SNR) of 42 dB. The center frequency, −6 dB bandwidth, and SNR of the LiNbO_3_ transducer were 105 MHz, 66%, and 44 dB, respectively. In order to compare and evaluate the transducers’ performances, an ultrasonic biomicroscopy (UBM) imaging on the fish eye was performed. The results showed that the LiNbO_3_ transducer had a better contrast resolution compared to the PMN-0.3PT transducer. The fabricated transducer showed an excellent performance with high-resolution corneal epithelium imaging of the experimental fish eye. These interesting findings are useful for the future biomedical implementation of the fabricated transducers in the field of high-resolution ultrasound imaging and diagnosis purpose.

## 1. Introduction

Ultrasonic transducers are important, well-known devices related to image acquisition in biomedical applications [1,2,3], corrosion inspection, and defecting surface breaking cracks in industry [4,5]. These devices are made from several piezoelectric materials, depending on their applications. Each type of piezoelectric material can have a certain frequency range depending on its characteristics. It was reported in previous studies by Nguyen et al. [6] that a 9 µm polyvinylidene fluoride (PVDF) film was successfully used to fabricate a 50 MHz focused transducer. Single-element poly (vinylidene fluoride trifluoroethylene) (P(VDF-TrEE)) film achieved approximately a 20 MHz frequency with a thickness of 25 µm [7]. Wong et al. [8] designed a 20 MHz 64-elements phased array transducer using a PMN-0.3PT single crystal, which supported biomedical applications. The reported lead-free Bi0.5Na0.5TiO_3_ composite film transducer achieved approximately a 98 MHz frequency with an 11 µm thickness [9]. According to Hsu et al. [10], a ~9 µm silver-doped PMN-PT-PZT membrane was used to fabricate a 225 MHz high-frequency needle transducer. By applying the cutting and filling method, Wang et al. [11] fabricated the PMN-PT/Epoxy 1–3 piezoelectric composite ultrasonic transducer with 42 MHz. The fabrication process [12,13] of a high-frequency transducer is very sophisticated and highly complicated.

The matching layer technique plays an important role in improving the transducer’s performance with a high bandwidth and reduces its attenuation. A significantly different impedance between the piezoelectric element and the ultrasound medium (e.g., water) causes a mismatch during energy transmission. In order to reduce the acoustic impedance mismatch between the two different impedances, a layer of matching material should be bonded on the front surface of the transducer. Over the last decade, many studies on associating a single matching layer with a quarter-wavelength thickness were reported [14]. Zhou et al. [15] developed a single matching layer technique with a quarter-wavelength thickness to improve the performance of transducers. Meanwhile, several researchers [16,17,18] reported a transducer performance with a double matching layer technique. The impedance and thickness (quarter-wavelength) of a material are the important characteristics of a conventional quarter-wavelength mechanism [18]. The impedance can be different due to the various mixing ratios of particles, powder, and polymers [19,20]. It is a challenge to attain both a specific acoustic impedance and precise thickness. Recently, a few studies discussed the new approach of the mass–spring matching layer for high-frequency transducers [21,22]. This method involved interfacing with metal–polymer layers for matching effects. The mass–spring layers were vapor-deposited on the front surface of the transducer with a controlled thickness. One advantage of the vacuum deposition method, compared to the conventional quarter-wavelength technique [16], is the optional selection of materials and their thicknesses. The mass–spring matching layer technique overcomes the limitations of the method of precisely lapping. Matching layers can be performed on a spherical surface in order to minimize energy loss.

Recently, it was found that the acoustic microparticle trapping technique is the most popular method for a single ultrasonic transducer [23]. In this technique, highly focused small transducers are used to capture a liquid particle without any contacts between the transducer and the microparticles. To execute this method with a smaller particle detection, a high-frequency transducer has to be applied to a higher spatial resolution. Moreover, small transducers offer more flexibility in small areas where the experiments can be performed [24]. It is worth noting that the beam diameter of the focused transducer becomes smaller when increasing the transducer’s frequency and larger when increasing the f-number. The f-number is a parameter of transducers that is defined by the ratio between the focal depth and the aperture size of the transducer. The small f-number and broad bandwidth created the high-resolution transducer, which forms a best B-scan image quality. A two-dimensional B-scan image was obtained by scanning the cross-sectional data of the object using a transducer and collecting pulse–echo lines in single-step size spacing. The B-scan displays the depth of different structures within the object that could be measured. During this scanning process, the transducer produces ultrasound waves that pass through the object. A portion of the ultrasound wave reflects the transducer and transmits the pulse–echo signal to the image processing unit to form an image.

Some previous studies [22,25,26] only applied the mass–spring technique on flat-surface transducers or lower 50 MHz focused transducers. The pulse–echo response method was used to test and evaluate the fabricated transducers, and the wire phantom experiment [27,28,29] was conducted to calculate the lateral and axial resolutions of the transducer and to demonstrate its performance. The fabrication and evaluation of focused needle transducers (100 MHz) was reported in this study. The aperture size of the transducer was designed to be 1.2 × 1.2 mm^2^, and the focal length of the transducer was 1.5 mm. The major difference was that the matching layer technique (mass–spring) was applied to a spherically shaped transducer. Furthermore, earlier studies reported that a 100 MHz self-focused ZnO transducer with a −6 dB bandwidth of 49% [30] was not able to display the lens of a zebrafish eye. Meanwhile, our current study verified the possibility of imaging a fish eye for medical imaging applications.

In our current study, we fabricated a high-frequency transducer based on the lead magnesium niobate-lead titanate (PMN-0.3PT) and lithium niobate (LiNbO_3_) single crystals. The structure of our reported study is as follows: in Section 2, the transducer design and fabrication are introduced; Section 3 demonstrates the transducer performances in biomedical applications with related discussions; and Section 4 provides conclusions and a summary of the transducer results.

## 2. Materials and Methods

### 2.1. Materials

To obtain a high frequency (100 MHz) of ultrasonic transducers, two types of piezoelectric materials—lithium niobate (LiNbO_3_) single crystal and lead magnesium niobate-lead titanate (PMN-0.3PT) single crystal—were selected. Due to the fact that these piezoelectric elements exhibit excellent electrical properties, they were suitable for fabricating a high-frequency transducer. The properties of these single crystals are shown in Table 1.

The properties of a 36° rotated Y-cut lithium niobite^1^ and PMN-0.3PT^2^ were determined by Boston Piezo-Optics (Bellingham, MA, USA) and Ceracomp (Seoul, Korea), respectively.

The ultrasound properties of the LiNbO_3_ element, including the electromechanical coupling coefficient (*k_t_*), relative clamped dielectric constant (*ε*^s^/*ε*_0_), density, and acoustic impedance were found to be much lower than those of the PMN-0.3PT element. The longitudinal wave velocity and Curie temperature (°C) of the LiNbO_3_ elements were significantly higher than those of the PMN-0.3PT element. In order to compare the characteristics of the two transducers, the fabrication process was performed as per the following stages.

### 2.2. Transducer Design

**Figure 1:**
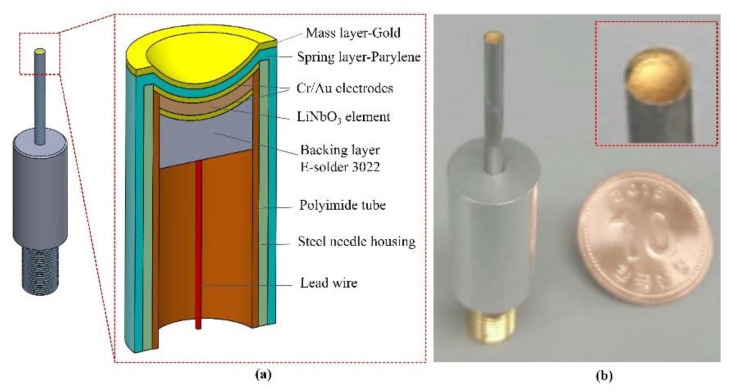
(**a**) A cross-sectional view of the focused needle transducer. (**b**) A digital photograph of the fabricated focused needle transducer.

Figure 1a illustrates the cross-sectional view of the focused needle transducer with all the major components used for fabrication. The piezoelectric element size was 1.2 × 1.2 mm^2^. A steel ball with a diameter of 3 mm was used to create a focal length of 1.5 mm. For small-sized transducers, a 14 G stainless-steel needle (Syringe needle, Huangshan, China) was used as the housing of the transducer. To support the experimental setup, the aluminum housing (diameter: 10 mm) was connected to the needle housing and an SMA connector (Mouser Electronics, Mansfield, TX, USA).

The developed transducer had three ports: two for mechanical functions, and one for electrical function [15,31]. The front and the back surfaces of the piezoelectric material served as mechanical ports. The front surface of the piezoelectric element indirectly touched the target through the load medium and connected to the negative pole of electric power through the housing and connector. The back surface of the piezoelectric material was connected to the positive pole of electrical power through the backing material [32] and connector. The electrical port transferred the electrical signal from the source to the piezoelectric element. To increase the sensitivity and the bandwidth of the transducer, the backing layer, the matching layer, and the lens materials needed to be connected to the piezoelectric elements. In addition, a conductive epoxy (E-solder 3022; Von Roll Isola Inc., New Haven, CT, USA) was used as a backing material with a sufficient attenuation to remove the backing echo during the two-time travel through the backing thickness (4 mm).

Owing to the big difference between the acoustic impedance of the piezoelectric material and the medium (e.g., water), in the case of high-frequency transducers, the compensation purpose based on matching layer was essential. To compensate for the acoustic impedance mismatch between the two different acoustic impedance, and to achieve better ultrasound signals, a matching layer was developed. However, to avoid the bonding layers in the conventional wavelength of the quarter-matching technique, the vacuum deposition method was used to deposit the matching layers, which is known as the mass–spring matching layers approach or metal–polymer matching layer technique. In the previous reports [21,22,33], the mass layer was one of the materials: copper, brass, or gold, and the spring layer was polyimide or parylene. In this present study, gold–parylene materials were selected because they could be easily vapor-deposited by the vacuum deposition system. 

The estimated resonance frequencies of two combined layers of mass and spring and the equivalent impedance (*Z_in_*) were calculated as follows [21]:

(1) $$f_{0}=\frac{1}{2\pi }\sqrt{\frac{\rho_{s}v_{s}^{2}/t_{s}}{\left(\rho_{m}t_{m}+0.4\rho_{s}t_{s}\right)}}$$

(2) $$Z_{in}=\frac{\left(\rho_{m}t_{m}+0.4\rho_{s}t_{s}\right)\rho_{s}v_{s}^{2}}{t_{s}Z_{l}}$$

where *f*_0_ denotes the resonance frequency; ρ*_m_* and ρ_s_ are the densities of the mass layer and the spring layer, respectively; *t_m_* and *t_s_* are the thicknesses of the mass layer and the spring layer, respectively; vs. represents the speed of sound in the spring layer; and *Z_in_* and *Z_l_* are the equivalent impedance and the load impedance, respectively.

Equation (1) revealed the relationship between two thicknesses at a specific working frequency, which represents the given thickness of the spring layer. The input impedance (*Z_in_*) was associated with the single quarter-wavelength (*λm*/4) acoustic impedance (*Z_λm_*_/4_) [31]:

(3) $$Z_{\lambda m/4}=\sqrt{Z_{in}Z_{l}}$$

Based on Equations (1)–(3), the equation to calculate the spring thickness can be expressed as Equation (4):

(4) $$t_{s}=\frac{\rho_{s}v_{s}^{2}}{2\pi f_{0}Z_{\lambda m/4}}$$

Combining Equations (1) and (4), the required mass layer thickness was calculated as follows:

(5) $$t_{m}=\frac{\rho_{s}v_{s}^{2}}{\rho_{m}t_{s}{\left(2\pi f_{0}\right)}^{2}}-\frac{0.4t_{s}\rho_{s}}{\rho_{m}}$$

Fei et al. [22] reported that, at the designed frequency (100 MHz), the equivalent impedance at the surface of the piezoelectric element was calculated with the following equation:

(6) $$Z_{in}=\sqrt[{3}]{Z_{p}^{2}Z_{l}}$$

where *Z_p_* and *Z_l_* represent the piezoelectric element impedance and load impedance, respectively.

The properties of the passive materials were selected in this study as shown in Table 2. In the specific case of LiNbO_3_ and PMN-0.3PT crystals, the equivalent acoustic impedances (*Z_in_*) were 12.11 and 12.61 *MRayl*, respectively. The approximate thicknesses of the gold and parylene layers are shown in Table 3.

The Krimholtz–Leedom–Matthaei (KLM) model was employed to estimate the parameters of the transducer. Based on the KLM model, the types and thicknesses of suitable matching materials could be determined, as shown in Table 3.

### 2.3. Fabrication Method

**Figure 2:**
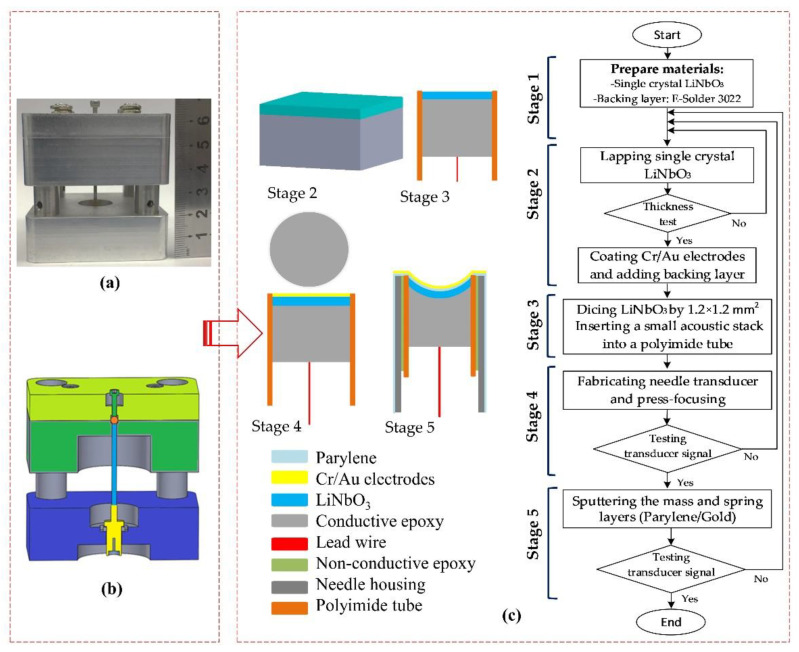
(**a**) A digital photograph of the press-fit system, (**b**) cross-sectional view of the press-fit system, and (**c**) the fabrication stages of press-focused needle transducer.

The five fabrication stages of the focused needle transducer used for their development are shown in Figure 2. Firstly, a single crystal of a 36° rotated Y-cut LiNbO_3_ (Boston Piezo-Optics, Bellingham, MA, USA) and a conductive epoxy (E-solder 3022; Von Roll Isola Inc., New Haven, CT, USA) with an acoustic impedance of 5.92 *MRayl* [23] were prepared as the piezoelectric material and backing material. In the second stage, a single-crystal LiNbO_3_ wafer was lapped down to 30 µm and polished to make a 100 MHz transducer. Then, a chrome/gold (50/100 nm) layer was sputtered on both sides of the LiNbO_3_ element. The lapping side at the rear surface was cast by a conductive epoxy of E-solder 3022 which is called a backing material. After conductive epoxy curing, the backing material was reduced to 2 mm in thickness. In the third stage, the sample was diced to a 1.2 × 1.2 mm^2^ post of the acoustic stack. A small acoustic stack was inserted into a polyimide tube (PI tubing; HnG Medical Incorporated, Richmond Hill, ON, Canada) with an inner diameter of 1.4 mm. Conductive epoxy was used to connect an electrical wire to the backing material. The acoustic stack with the polyimide tube was placed in housing using nonconductive epoxy (EPO-TEK 301; Epoxy Technology, Inc., Billerica, MA, USA). The SMA connector was connected to the acoustic stack through the lead wire and housing. The press-fit system (Figure 2a) was used to form a spherically shaped piezoelectric element at 90°. Conductive epoxy was used to connect the front surface and housing, forming the ground connection. In the fifth stage, an E-beam evaporator system (FC-2000; Temescal, Livermore, Canada) was used to develop the mass–spring matching layer. In this stage, 3 µm parylene and 200 nm gold were vapor-deposited on the front face of the transducer to assist as acoustic matching layers. According to the flowchart in Figure 2c, a completed focused needle transducer passed 5 stages. These testing steps in stages 2, 4, 5 helped to ensure that the desired parameters were achieved. Therefore, precise assembly skills are very important to obtain quality products. A small number of finished products that meet the specifications were used in research experiments. A digital photograph of the fabricated transducer is shown in Figure 1b.

The transducer product, successfully assembled in stage 4, was used for ultrasound imaging experiments. Before performing the mass–spring matching layer technique, an ultrasound biomicroscopy image (UBM) of the fish eye obtained with the LiNbO_3_ transducer was conducted. Similar to the stage 4, after performing the mass–spring matching layer technique, a UBM of the fish eye was created with the LiNbO_3_ transducer. These two UBMs of the same fish eye sample were performed with the same transducer. Therefore, the function difference between the transducer before and after the matching layer process could be compared through two UBMs.

For the PMN-0.3PT transducer, the sample was purchased from Ceracomp, Seoul, Korea. With extreme care and patience, the piezoelectric element was lapped down to 10 µm in thickness, and the size of the piezoelectric post was made to be 1.2 × 1.2 mm^2^. The fabrication process of the PMN-0.3PT transducer was similar to that of the LiNbO_3_ transducer.

### 2.4. Experimental Setup

**Figure 3:**
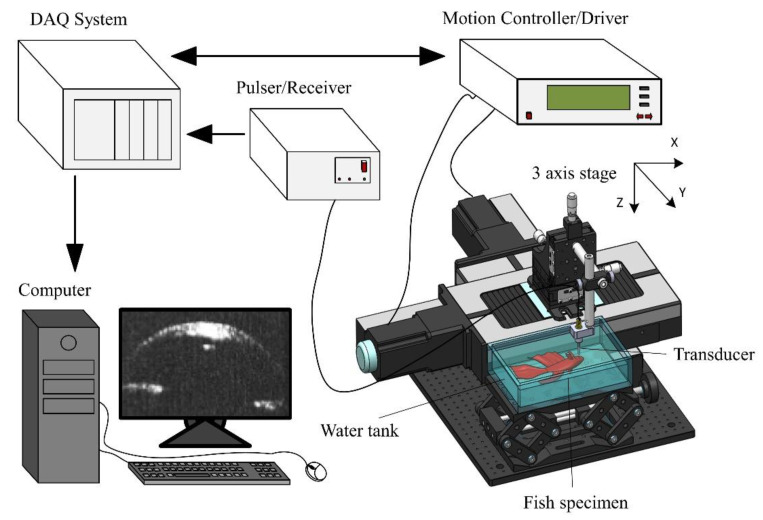
Schematic diagram of the experimental setup for ultrasound imaging using transducer.

The experimental system for testing and scanning the data is illustrated in Figure 3. An electrical impulse with a repetition rate of 200 Hz at 50 Ω damping and 3 μJ energy per pulse was excited from a computer-controlled remote (DPR500; JSR Ultrasonics, Pittsford, NY, USA) pulser/receiver for transducer operation. One glass plate was placed at the focal depth as a target to measure the pulse–echo and frequency spectra of the transducer. In order to obtain the reflected waveform, a 500 MHz bandwidth receiver with a high-pass filter of 5 MHz and a low-pass filter of 500 MHz was used. The received raw data were digitized at a high-speed sampling frequency of 500 million samples per second. The echo signals were digitized using an 8-bit digitizer (NI PCI-5153EX; National Instruments, Austin, TX, USA).

Stepper motors (UE63PP; Newport Corporation, Irvine, CA, USA) are driven using a universal motion controller/driver (ESP300; Newport Corporation) to control the movement of the transducer. In order to obtain the B-scan, a computer-controlled scanning stage and a developed LabView (LabView 2014; National Instruments) program were utilized to control all of the processes mentioned above.

To determine the spatial resolution of the transducer through a B-scan image, an 8 µm single-wire phantom experiment was performed. Two types of developed focused needle transducers were utilized to conduct the experiment on the same fish eye specimen. The RF data were used to generate these images using a logarithmic compression algorithm with a 40 dB dynamic range. To display the experimental images, data were processed using software based on MATLAB (Version 2013a; MathWorks, Natick, MA, USA).

## 3. Results and Discussion

**Figure 4:**
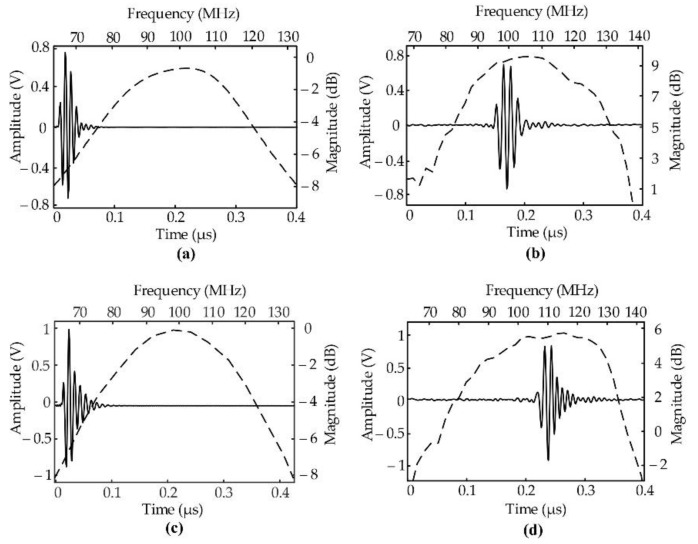
Pulse–echo response (solid line) and frequency spectra (dashed line) for a focused needle transducer with 1.5 mm of focal length. (**a**) Simulated signals and (**b**) measured signals of 103 MHz PMN-0.3PT transducer with 52 % BW at −6 dB and a 10 µm thickness. (**c**) Simulated signals and (**d**) measured signals of 105 MHz LiNbO_3_ with 66% BW at −6 dB and a 28 µm thickness.

Figure 4 displays the simulated results from the BioSono KLM (Figure 4a,c) and measured characteristics of the two transducers. The PMN-0.3PT transducer had a measured center frequency of 103 MHz and a −6 dB bandwidth of 52% (Figure 4b). The measured center frequency of the LiNbO_3_ transducer was 105 MHz and its −6 dB bandwidth was 66% (Figure 4d). The measured center frequencies were a little higher than the simulated frequency. Because of the thickness vibration mode of the piezoelectric, the thinner piezoelectric creates a higher frequency transducer. The simulation of 100 MHz LiNbO_3_ transducers was 30 µm in thickness. The thickness of LiNbO_3_ was lapped down to 28 µm; therefore, the center frequency was increased to 105 MHz.

**Figure 5:**
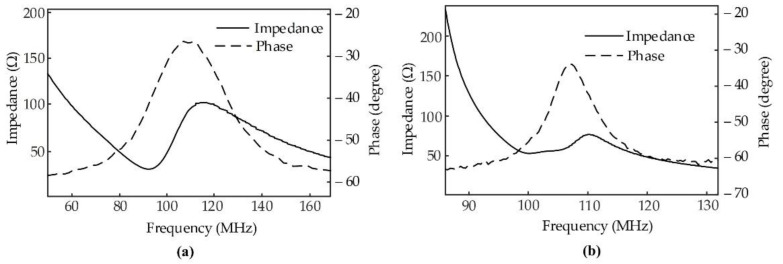
Measured electrical impedance magnitude (solid line) and phase angle (dashed line) (**a**) 103 MHz PMN-0.3PT transducer and (**b**) 105 MHz LiNbO_3_ transducer.

The electrical impedance (magnitude and phase) of the transducers is shown in Figure 5 and was measured by an Agilent Keysight 4396B impedance analyzer (Agilent Technologies, Santa Clara, CA, USA). From the electrical impedance graph, the resonance frequencies and impedances of the transducers were determined. In order to evaluate the electromechanical efficiency of the transducer, the electromechanical coupling coefficient (*k_t_*) of the thickness vibration mode was calculated as [34]:

(7) $$k_{t}=\sqrt{\frac{\pi }{2}\frac{f_{r}}{f_{a}}tan\left(\frac{\pi }{2}\frac{f_{a}-f_{r}}{f_{a}}\right)}$$

where, *f_r_* and *f_a_* are the resonant and anti-resonant frequencies of the thickness vibration mode, respectively. Figure 5 indicates the measured electrical impedance magnitude and phase plots for two types of transducers in air, showing a clear single-thickness mode resonance. The resonant and anti-resonant frequencies of the PMN-0.3PT transducer are 92 and 114 MHz, and those of the LiNbO_3_ transducer are 99 and 110 MHz, respectively. From Equation (7), electromechanical coupling coefficients were calculated to be 0.62 and 0.47 for the PMN-0.3PT and LiNbO_3_, respectively. These values are in agreement with reports from the suppliers.

In addition, the signal-to-noise ratio (*SNR*) was calculated to evaluate the mass–spring matching technique, which is capable of improving the efficiency of signal transfer [35]:

(8) $$SNR=20\log_{10}\left(\frac{\max\left(E\right)}{\sigma_{noise}}\right)$$

where *E* and *σ_noise_* denote the envelope of the echo signal and the standard deviation of the system noise, respectively.

**Figure 6:**
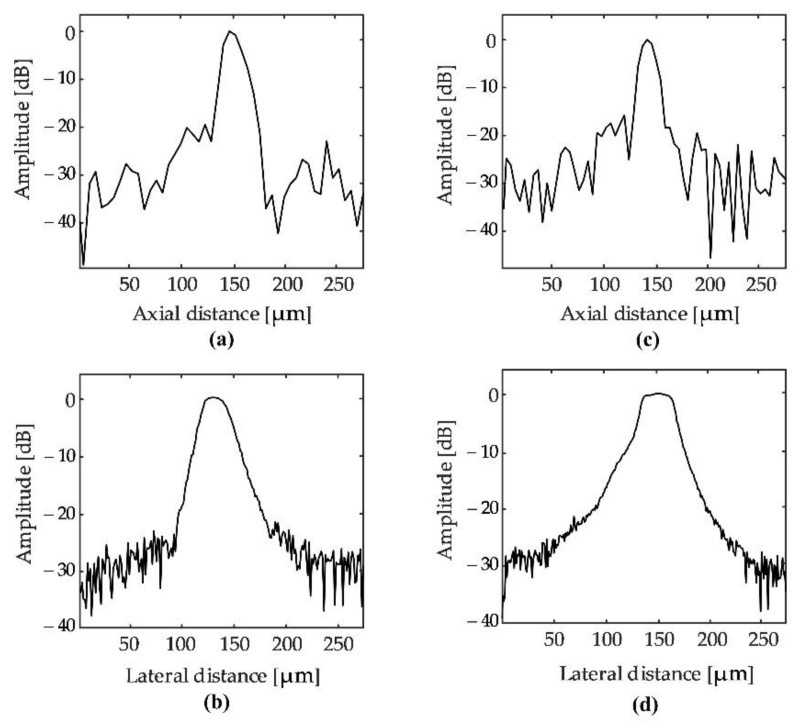
(**a**,**b**) Measured axial and lateral resolution of the PMN-0.3PT transducer. (**c**,**d**) Measured axial and lateral resolution of the LiNbO_3_ transducer.

Figure 6 shows the beam profile of the transducers. In the 100 MHz range, the measured axial resolution and lateral resolution of the LiNbO_3_ transducer were higher than those of the PMN-0.3PT transducer. The 105 MHz LiNbO_3_ transducer had a measured axial resolution of 16 µm and a lateral resolution of 45 µm by full width at half maximum (−6 dB) of the corresponding cross-sectional profile and wire phantom. Similarly, for the PMN-0.3PT transducer, the measured axial and lateral resolutions were 18 µm and 48 µm, respectively.

Table 4 provides a summary of the measured transducer characteristics. Regarding the features of these two transducers, the LiNbO_3_ transducer had a higher sensitivity and a wider broadband compared to the PMN-0.3PT transducer. The lateral resolutions of PMN-0.3PT and LiNbO_3_ transducers detected a spatial point target at full width and half maximum (FWHM, −6dB), which was consistent with the theoretical, lateral resolution of 100 MHz transducers, equal to 19.25 µm (f-number × wavelength at center frequency).

In a previous study conducted by Chunlong Fei et al. [22], a 100 MHz lithium niobate flat transducer was developed with the mass–spring matching technique, which increased the transducer bandwidth from 31.2% to 58.3%. The study only made a comparison between the pulse–echo experiment and the transducer before and after the matching layers. The application of ultrasound imaging was not used in this study. In our study, the 105 MHz lithium niobate focused needle transducer increased the bandwidth from 36% to 66%, corresponding to the mass–spring matching layer functions and no matching. To demonstrate the feasibility of fish eye images, the mass–spring matching approach was applied to the spherical shape of the different focused needle transducers and demonstrated the feasibility of transducer performances by UBM fish eye imaging.

**Figure 7:**
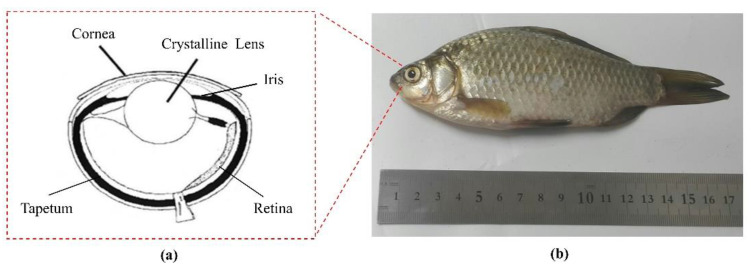
(**a**) Structure of the fish eye. (**b**) A digital photograph of the fish specimen.

To demonstrate the performance of the transducer, a B-scan image of a fish eye (Crucian carp) was obtained by performing an experiment. Figure 7 illustrates the structure of the eye and a digital photograph of the fish specimen. All crucian carp studies were performed in compliance with IACUC protocol No. PKNUIACUC-2019-10 approved by Pukyong National University. In this experimental study, only a single eye of a fish specimen was used to conduct the B-scan imaging. The fish specimen length was approximately 170 mm, and its eye diameter was approximately 7 mm. The fish specimen was fixed at the bottom of a water bath, and the eye was adjusted at the depth of field of the transducer to obtain the best image quality.

**Figure 8:**
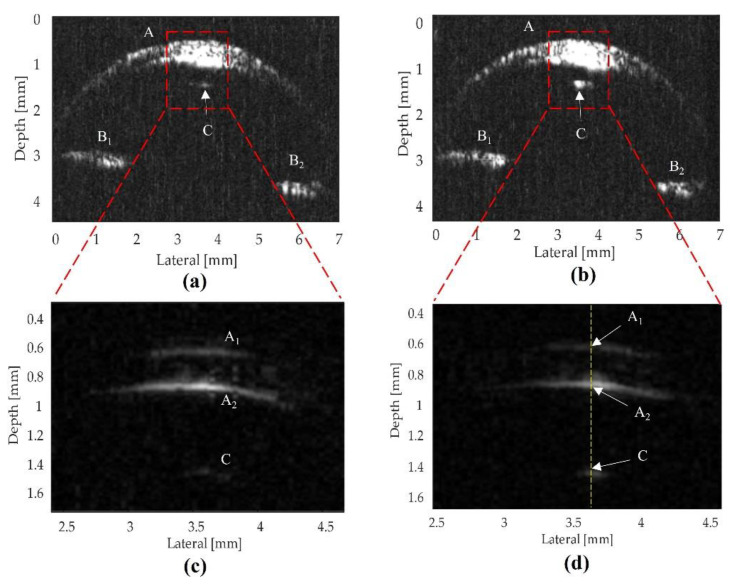
Before performing the mass–spring matching layer technique, an ultrasound biomicroscopy image (UBM) of the fish eye obtained with (**a**) the 103 MHz PMN-0.3PT transducer and (**b**) the 105 MHz LiNbO_3_ transducer. (**c**,**d**) Enlarged view of the center of the cornea using the filter to remove the lower-frequency signal. The cornea (A), upper cornea surface (A_1_), lower cornea surface (A_2_), left iris (B_1_), right iris (B_2_), and lens (C) are visible in both images.

Figure 8 displays the B-scan images of the fish eye obtained by two transducers. The image shows the shapes of the cornea, iris, and lens of the fish eye specimen. The experimental image shows the top depth surface (A_1_ = 0.625 mm), the bottom surface (A_2_ = 0.883 mm), and the lens (C = 1.456 mm). Therefore, the thickness of the cornea was calculated as 0.258 mm, and the distance between the lower surface of the cornea and the lens surface was 0.573 mm. From these data, the ultrasound penetration depth from the top surface of the cornea was measured to be 0.831 mm.

The comparison of the ultrasound biomicroscopy images of the fish eye taken by the two different transducers was conducted as follows. In the absence of the mass–spring matching layers, the image from the LiNbO_3_ transducer (Figure 8b) was brighter than the image (Figure 8a). The fish eye lens image (Figure 8c) captured by the LiNbO_3_ transducer (Figure 8b) had a higher contrast resolution than that of the image (Figure 8a). It was revealed that the sensitivity of the ultrasound signal from the LiNbO_3_ transducer was higher compared to PMN-0.3PT transducer.

**Figure 9:**
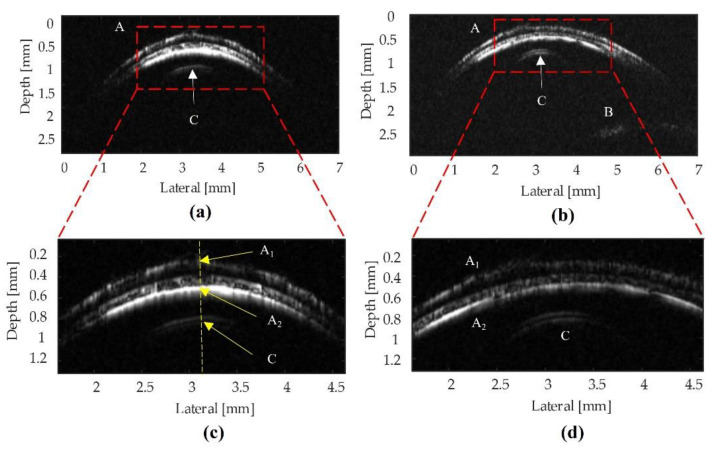
After performing the mass–spring matching layer technique, a UBM of the fish eye obtained was obtained with (**a**) the 103 MHz PMN-0.3PT transducer and (**b**) the 105 MHz LiNbO_3_ transducer. (**c**,**d**) Enlarged view of the center of the cornea. The cornea (A), upper cornea surface (A_1_), lower cornea surface (A_2_), iris (B), and lens (C) are visible.

In addition, the authors also found a significant difference between the matching layer and unmatching layer transducers, which are represented in Figure 8 and Figure 9. If there was a mass–spring matching layer (Figure 9), the layers in areas A_1_, A_2_ and C were clearer, as shown in Figure 8. The fish eye lens in the unmatching layer showed only a point (Figure 8c), whereas, in the matching layer, the eye lens showed a clear view, as an arch (Figure 9c). Transducers with matching layers displayed a high-resolution micrograph with separate cornea layers and decreased background noise levels due to their enhanced spatial resolution. Therefore, the high matching performance of high-frequency transducers produced high SNR (42–44 dB), which was suitable for high-resolution medical image applications.

## 4. Conclusions

In this study, high-frequency (above 100 MHz) focused needle transducers were successfully fabricated using PMN-0.3PT and LiNbO_3_ single crystals with a mass–spring matching layer. The mass–spring matching layer approach helped to avoid the limitations of the conventional quarter-wavelength method for developing high-frequency transducers. The novelty of this work is its unique fabrication technique employing a mass–spring matching method. Mass–spring layers were vapor-deposited on a spherical surface with a very thin layer of parylene (1.8 µm) and gold (200 nm), which ensured a uniform level for the whole sphere. Other reported studies were only applied on a plane surface (unfocused transducer). In this study, the PMN-0.3PT transducer achieved a center frequency of 103 MHz, with a 52% bandwidth at −6 dB and SNR of 42 dB. The measured center frequency, −6 dB bandwidth, and SNR achieved by the LiNbO_3_ transducer were 105 MHz, 66%, and 44 dB, respectively. The B-scan image of the fish eye was obtained by scanning the transducer along the X-axis over the fish eye specimen, which produced a cross-sectional view of the fish eye. The B-scan images showed the measured depths of different structures within the fish eye. The high-resolution ultrasonic biomicroscopy images of the fish eye were obtained using a fabricated transducer, which clearly showed the two layers of the corneal epithelium of the fish eye. These interesting results encourage further research and potential applications of the transducer for more critical biomedical applications. The findings of this study validated the potential application of this fabrication technique.

## Figures and Tables

**Table 1:** Important material properties of 36° rotated Y-cut lithium niobate^1^ and PMN-0.3PT^2^.

Property	LiNbO_3_	PMN-0.3PT
Electromechanical coupling coefficient (*k_t_*)	0.49	0.57
$\mathrm{Relative}\;\mathrm{clamped}\;\mathrm{dielectric}\;\mathrm{constant}\;(\varepsilon^{s}$$/\varepsilon_{0}$)	39	870
Density (g/cm^3^)	4.64	7.98
Longitudinal wave velocity (m/s)	7340	4529
Acoustic impedance (*MRayl*)	34.0	36.1
Curie temperature (°C)	1150	130

**Table 2:** Materials properties selected in the transducer design [22].

Materials	Function	Velocity of Sound (m/s)	Density (kg/m^3^)	Acoustic Impedance (*MRayl*)
Water	Front load	1540	1000	1.54
Gold	Matching layer	3240	19700	63.8
Parylene	Matching layer	2350	1100	2.58
E-Solder 3022	Conductive backing	1850	3200	5.92

**Table 3:** Predicted parameters on the basis of the KLM model for the transducer design.

Predicted Parameters	LiNbO_3_	PMN-0.3PT
Frequency (MHz)	100	100
Aperture size (mm × mm)	1.2 × 1.2	1.2 × 1.2
Piezoelectric thickness (µm)	30	10
Focal length (mm)	1.5	1.5
1st matching layer thickness of parylene (µm)	3	1.8
2nd matching layer thickness of gold (nm)	200	550

**Table 4:** Measured transducer characteristics.

Properties	LiNbO_3_	PMN-0.3PT
Aperture size (mm × mm)	1.2 × 1.2	1.2 × 1.2
Focal length (mm)	1.5	1.5
Center frequency f_c_ (MHz)	103	105
−6 dB Bandwidth (%)	52	66
Impedance Z (Ω)	44	46
Resonant frequency fr (MHz)	92	99
Anti-resonant frequency fa (MHz)	114	110
Electromechanical coupling coefficient kt	0.62	0.47
Axial resolution (µm)	18	16
Lateral resolution (µm)	48	45
Signal-to-noise ratio (SNR) (dB)	42	44

## References

[1] Liu Y., Li D., Yuan Z. (2016). Photoacoustic tomography imaging of the adult zebrafish by using unfocused and focused high-frequency ultrasound transducers. Appl. Sci..

[2] Lee J., Moon J.-Y., Chang J.H. (2018). A 35 MHz/105 MHz dual-element focused transducer for intravascular ultrasound tissue imaging using the third harmonic. Sensors.

[3] Nguyen T.P., Nguyen V.T., Mondal S., Pham V.H., Vu D.D., Kim B.-G., Oh J. (2020). Improved depth-of-field photoacoustic microscopy with a multifocal point transducer for biomedical imaging. Sensors.

[4] Khalili P., Cawley P. (2018). The choice of ultrasonic inspection method for the detection of corrosion at inaccessible locations. NDT E Int..

[5] Peng C., Bai L., Zhang J., Drinkwater B.W. (2018). The sizing of small surface-breaking fatigue cracks using ultrasonic arrays. NDT E Int..

[6] Nguyen T.P., Truong N.T.P., Bui N.Q., Nguyen V.T., Hoang G., Choi J., Phan T.T.V., Pham V.H., Kim B.-G., Oh J. (2019). Design, Fabrication, and Evaluation of Multifocal Point Transducer for High-Frequency Ultrasound Applications. Sensors.

[7] Jeong J.S., Kirk Shung K. (2013). Improved fabrication of focused single element P(VDF–TrFE) transducer for high frequency ultrasound applications. Ultrasonics.

[8] Wong C.-M., Chen Y., Luo H., Dai J., Lam K.-H., Chan H.L.-W. An ultrawide bandwidth high frequency phased-array ultrasound transducer fabricated using the PMN-0.3 PT single crystal. Proceedings of the 2016 IEEE International Ultrasonics Symposium (IUS).

[9] Yan X., Ji H., Lam K.H., Chen R., Zheng F., Ren W., Zhou Q., Shung K.K. (2013). Lead-free BNT composite film for high-frequency broadband ultrasonic transducer applications. IEEE Trans. Ultrason. Ferroelectr. Freq. Control.

[10] Hsu H.-S., Benjauthrit V., Wei Q., Huang Y., Zhou Q., Shung K.K. (2013). Silver doped 0.9 PMN-PT-0.1 PZT composite films for very high frequency ultrasonic transducer applications. Appl. Phys. A.

[11] Wang J., Chen M., Zhao X., Wang F., Tang Y., Lin D., Luo H. (2021). Fabrication and high acoustic performance of high frequency needle ultrasound transducer with PMN-PT/Epoxy 1-3 piezoelectric composite prepared by dice and fill method. Sens. Actuators A Phys..

[12] Wang X.-B., He L.-M., Liu W.-J., Song S.-R., Xu W.-J., Cheng Q., Riaud A., Ren J.-Y., Zhou J. Development of PZT-based 18 MHz 2D pMUT array with PDMS waveguide. Proceedings of the 2020 IEEE International Ultrasonics Symposium (IUS).

[13] Wang X.-B., He L.-M., Ma Y.-C., Liu W.-J., Xu W.-J., Ren J.-Y., Riaud A., Zhou J. (2021). Development of broadband high-frequency piezoelectric micromachined ultrasonic transducer array. Sensors.

[14] Lam K.H., Ji H.F., Zheng F., Ren W., Zhou Q., Shung K.K. (2013). Development of lead-free single-element ultrahigh frequency (170–320 MHz) ultrasonic transducers. Ultrasonics.

[15] Zhou Q., Lam K.H., Zheng H., Qiu W., Shung K.K. (2014). Piezoelectric single crystal ultrasonic transducers for biomedical applications. Prog. Mater. Sci..

[16] Wang Y., Tao J., Guo F., Li S., Huang X., Dong J., Cao W. (2018). Magnesium Alloy Matching Layer for High-Performance Transducer Applications. Sensors.

[17] Yoon S., Kim M.G., Williams J.A., Yoon C., Kang B.J., Cabrera-Munoz N., Shung K.K., Kim H.H. (2015). Dual-element needle transducer for intravascular ultrasound imaging. J. Med. Imaging.

[18] Wong C.-M., Chen Y., Luo H., Dai J., Lam K.-H., Chan H.L.-W. (2017). Development of a 20-MHz wide-bandwidth PMN-PT single crystal phased-array ultrasound transducer. Ultrasonics.

[19] Park J.-H., Lee S.-M., Park J., Lee H.J., Paik K.-W. (2020). Acoustic matching layer films using B-stage thermosetting polymer resins for Ultrasound Transducer Applications. IEEE Trans. Ultrason. Ferroelectr. Freq. Control.

[20] Wong C.-M., Chan S.-F., Wu W.C., Suen C.-H., Yau H.-M., Wang D.Y., Li S., Dai J.Y. (2021). Tunable high acoustic impedance alumina epoxy composite matching for high frequency ultrasound transducer. Ultrasonics.

[21] Brown J.A., Sharma S., Leadbetter J., Cochran S., Adamson R. (2014). Mass-spring matching layers for high-frequency ultrasound transducers: A new technique using vacuum deposition. IEEE Trans. Ultrason. Ferroelectr. Freq. Control.

[22] Fei C., Ma J., Chiu C.T., Williams J.A., Fong W., Chen Z., Zhu B., Xiong R., Shi J., Hsiai T.K. (2015). Design of matching layers for high-frequency ultrasonic transducers. Appl. Phys. Lett..

[23] Hsu H.-S., Zheng F., Li Y., Lee C., Zhou Q., Kirk Shung K. (2012). Focused high frequency needle transducer for ultrasonic imaging and trapping. Appl. Phys. Lett..

[24] Li X., Wu W., Chung Y., Shih W.Y., Shih W.-H., Zhou Q., Shung K.K. (2011). 80-MHz intravascular ultrasound transducer using PMN-PT free-standing film. IEEE Trans. Ultrason. Ferroelectr. Freq. Control.

[25] Yang X., Fei C., Li D., Sun X., Hou S., Chen J., Yang Y. (2020). Multi-layer polymer-metal structures for acoustic impedance matching in high-frequency broadband ultrasonic transducers design. Appl. Acoust..

[26] Lee J., Jang J., Chang J.H. (2016). Oblong-shaped-focused transducers for intravascular ultrasound imaging. IEEE Trans. Biomed. Eng..

[27] Bui N.T., Nguyen T.M.T., Dinh T.T.N., Bui Q.C., Vo T.H., Phan D.T., Park S., Choi J., Kang Y.-H., Kim B.-G. (2020). Design of a Multichannel Pulser/Receiver and Optimized Damping Resistor for High-Frequency Transducer Applied to SAM System. Appl. Sci..

[28] Truong N.T.P., Choi J., Park S., Ly C.D., Cho S.-W., Mondal S., Lim H.G., Kim C.-S., Oh J. (2021). Ultra-widefield photoacoustic microscopy with a dual-channel slider-crank laser-scanning apparatus for in vivo biomedical study. Photoacoustics.

[29] Zhang Z., Hong W.-C. (2019). Electric load forecasting by complete ensemble empirical mode decomposition adaptive noise and support vector regression with quantum-based dragonfly algorithm. Nonlinear Dyn..

[30] Cannata J.M., Williams J.A., Zhou Q.F., Sun L., Shung K.K., Yu H., Kim E.S. (2008). Self-focused ZnO transducers for ultrasonic biomicroscopy. J. Appl. Phys..

[31] Zhou Q., Lau S., Wu D., Kirk Shung K. (2011). Piezoelectric films for high frequency ultrasonic transducers in biomedical applications. Prog. Mater. Sci..

[32] Kuscer D., Bustillo J., Bakarič T., Drnovšek S., Lethiecq M., Levassort F. (2020). Acoustic properties of porous lead zirconate titanate backing for ultrasonic transducers. IEEE Trans. Ultrason. Ferroelectr. Freq. Control.

[33] Toda M., Thompson M. (2012). Detailed investigations of polymer/metal multilayer matching layer and backing absorber structures for wideband ultrasonic transducers. IEEE Trans. Ultrason. Ferroelectr. Freq. Control.

[34] Hou S., Yang X., Fei C., Sun X., Chen Q., Lin P., Li D., Yang Y., Zhou Q. (2018). Fabrication of PMN-PT/Epoxy 2–2 Composite Ultrasonic Transducers and Analysis Based on Equivalent Circuit Model. J. Electron. Mater..

[35] Yoon C., Kim G.-D., Yoo Y., Song T.-K., Chang J.H. (2013). Frequency equalized compounding for effective speckle reduction in medical ultrasound imaging. Biomed. Signal Processing Control.

